# Michigan Model for Health^TM^ Learning to Enhance and Adapt for Prevention (Mi-LEAP): protocol of a pilot randomized trial comparing Enhanced Replicating Effective Programs versus standard implementation to deliver an evidence-based drug use prevention curriculum

**DOI:** 10.1186/s40814-022-01145-6

**Published:** 2022-09-10

**Authors:** Andria B. Eisman, Lawrence A. Palinkas, Christine Koffkey, Todd I. Herrenkohl, Umaima Abbasi, Judy Fridline, Leslie Lundahl, Amy M. Kilbourne

**Affiliations:** 1 Community Health, Division of Kinesiology, Health and Sport Studies, College of Education, Wayne State University, 2153 Faculty/Administration Building, 656 West Kirby, Detroit, MI 48202 USA; 2 Center for Health and Community Impact, College of Education, Wayne State University, Detroit, MI 48202 USA; 3 Suzanne Dworak-Peck School of Social Work, University of Southern California, Los Angeles, CA 90089 USA; 4 School of Social Work, University of Michigan, Ann Arbor, MI 48109 USA; 5 Department of Health Behavior and Health Education, School of Public Health, University of Michigan, Ann Arbor, MI 48109 USA; 6 Genesee Intermediate School District, Flint, MI 48507 USA; 7 School of Medicine, Wayne State University, Detroit, MI 48201 USA; 8 Department of Learning Health Sciences, University of Michigan Medical School, Ann Arbor, MI USA; 9 Health Services Research and Development, Veterans Health Administration, U.S. Department of Veterans Affairs, Washington, D.C., USA

**Keywords:** Implementation science, Prevention, Adolescents, Drug use disorders, Adverse childhood experiences, Costs, Cost-effectiveness

## Abstract

**Background:**

School-based drug use prevention programs have demonstrated notable potential to reduce the onset and escalation of drug use, including among youth at risk of poor outcomes such as those exposed to trauma. Researchers have found a robust relationship between intervention fidelity and participant (i.e., student) outcomes. Effective implementation of evidence-based interventions, such as the Michigan Model for Health^TM^ (MMH), is critical to achieving desired public health objectives. Yet, a persistent gap remains in what we know works and how to effectively translate these findings into routine practice. The objective of this study is to design and test a multi-component implementation strategy to tailor MMH to meet population needs (i.e., students exposed to trauma), and improve the population-context fit to enhance fidelity and effectiveness.

**Methods:**

Using a 2-group, mixed-method randomized controlled trial design, this study will compare standard implementation versus Enhanced Replicating Effective Programs (REP) to deliver MMH. REP is a theoretically based implementation strategy that promotes evidence-based intervention (EBI) fidelity through a combination of EBI curriculum packaging, training, and as-needed technical assistance and is consistent with standard MMH implementation. Enhanced REP will tailor the intervention and training to integrate trauma-informed approaches and deploy customized implementation support (i.e., facilitation). The research will address the following specific aims: (1) design and test an implementation strategy (Enhanced REP) to deliver the MMH versus standard implementation and evaluate feasibility, acceptability, and appropriateness using mixed methods, (2) estimate the costs and cost-effectiveness of Enhanced REP to deliver MMH versus standard implementation.

**Discussion:**

This research will design and test a multi-component implementation strategy focused on enhancing the fit between the intervention and population needs while maintaining fidelity to MMH core functions. We focus on the feasibility of deploying the implementation strategy bundle and costing methods and preliminary information on cost input distributions. The substantive focus on youth at heightened risk of drug use and its consequences due to trauma exposure is significant because of the public health impact of prevention. Pilot studies of implementation strategies are underutilized and can provide vital information on designing and testing effective strategies by addressing potential design and methods uncertainties and the effects of the implementation strategy on implementation and student outcomes.

**Trial registration:**

NCT04752189—registered on 8 February 2021 on ClinicalTrials.gov PRS

## Background

School-based universal prevention interventions have demonstrated notable potential to reduce the onset and escalation of drug use and mental health problems, including among youth exposed to trauma, marginalization, and socioeconomic disadvantage [1, 2]. Universal prevention interventions, also referred to as Tier 1, are delivered to an entire population regardless of risk [3]. These interventions can have a lasting impact on youth by reducing or preventing multiple interrelated outcomes (e.g., drug use and poor mental health) that share common risk factors [4–6]. School-based prevention can also reach large populations of young people, including those underserved in other settings [7]. Thus, schools are a critically important setting in which to support well-being and mitigate the effects of risk exposure among children and youth. Tier 1 prevention that is responsive to population needs offers a promising opportunity to reduce the short- and long-term consequences of exposure to stress and adversity, including substance abuse, the development of substance use disorders, mental illness, and academic failure, by enhancing resilience, providing a supportive context, and avoiding stigmatization and retraumatization [1, 8].

Recent research indicates that trauma exposure is pervasive among youth. An estimated 30.5% of youth ages 12–17 are exposed to multiple (2 or more) Adverse Childhood Experiences or ACEs [9]. ACEs are potentially traumatic events that occur during childhood including abuse, neglect, witnessing violence, parental substance abuse, and mental health problems [9, 10]. School-based, trauma-informed interventions represent a promising way to mitigate the impact of exposure to adversity on children and youth, especially given the reach of universal prevention and the high prevalence of trauma exposure in the general population [1]. Researchers have found higher rates of ACEs, other trauma, and toxic stress exposure among youth experiencing marginalization and socioeconomic disadvantage [11].

**Fig. 1:**
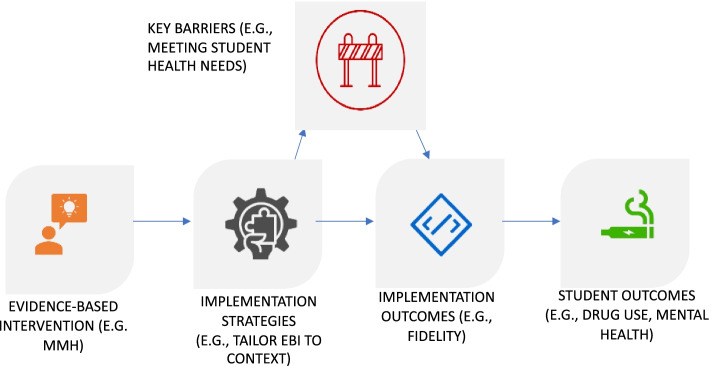
Conceptual model for applying implementation strategies to evidence-based interventions (EBIs), adapted from Proctor et al. [19]; Lyon & Bruns [20]

Taken together, this research indicates that school-based universal prevention would benefit from incorporating approaches to meet the needs of trauma-exposed youth. By incorporating trauma-informed approaches, teachers and other school professionals can reduce the risk of additional adversity exposure and retraumatization and strengthen factors that support resilience [12, 13]. Evidence-based interventions (EBIs), however, have rarely been designed to remain responsive to student needs, such as trauma exposure [14]. As a result, EBIs frequently fail to achieve desired public health outcomes, including among those who would most benefit [15, 16]. Researchers suggest that the public health impact of EBIs can be improved by addressing key determinants (or barriers) and facilitators of successful implementation (see Fig. 1). this would enhance the adoption, delivery, and sustainment of EBIs, as well as bridge the sizable gap between knowing which prevention strategies work and effectively translating such strategies into routine practice [17, 18]. Designing and deploying implementation strategies for existing universal prevention EBIs, such as the Michigan Model for Health^TM^ (MMH), offers an efficient way to address key barriers to implementation, meet population needs, and achieve public health objectives.

MMH is a theoretically-based, universal prevention curriculum that has demonstrated efficacy in randomized trials in reducing substance use and improving mental health outcomes among high school students [21, 22]. The curriculum is grounded in social cognitive theory [23] and the health belief model [24]. Our current study focuses on three core units of the MMH curriculum: the foundational skills unit, social and emotional health, and alcohol, tobacco, and other drugs. The MMH curriculum is recognized as an evidence-based intervention by CASEL (the Collaborative on Academic and Social and Emotional Learning) and is aligned with Michigan and National (USA) Health Education standards [25, 26]. The curriculum is widely adopted across Michigan, with 91% of health teachers using MMH [27]. Yet, similar to other EBIs, MMH is infrequently delivered with fidelity [28]. A statewide study found that 58% of educators failed to meet state-designated MMH fidelity standards [27] (i.e., delivering 80% or more of the curriculum); this is even higher among schools in economically challenged communities, with 73% not meeting state fidelity standards [29].

Replicating effective programs (REP) is a well-suited implementation strategy for school-based prevention. REP is a multi-component strategy used in community settings, including schools, focused on enhancing fit between the intervention and context while maintaining fidelity to core EBI functions. It is based on the CDC’s research-to-practice framework [30, 31] and guided by social cognitive [32] and diffusion of innovations theories [33]. REP is a low-level strategy that is consistent with the standard implementation of the MMH curriculum and includes three primary components: curriculum packaging materials, teacher training, and as-needed technical assistance (Table 1). As REP is not always sufficient to effectively implement complex behavioral interventions [34], researchers developed Enhanced REP that includes tailoring of curriculum materials, tailored training, and ongoing provider consultation, or Facilitation. Facilitation is based on the integrated Promoting Action on Research Implementation in Health Services (iPARIHS) framework, to provide more intensive implementation support [34, 35]. Researchers have found enhanced program **uptake** among clinical sites deploying multi-component implementation strategies such as Enhanced REP [36]. This additional tailoring and support can aid in mitigating barriers, enhancing intervention-context fit, and ultimately achieving desired public health outcomes [14]. Additionally, the cost of implementation fundamentally influences program delivery in schools, which often have competing demands and carefully allocated resources [7, 37]. To date, most economic evaluation has focused on intervention costs and *not* the costs of implementation strategies required to deploy and sustain them [37]. Systematic examination of costs and outcomes for multi-component implementation strategies is vital for scale-up and sustainability in community settings [37, 38].

**Table 1:** Standard implementation and Enhanced Replicating Effective Programs (Enhanced REP) components for drug use prevention intervention implementation (adapted from Kilbourne et al. [39])

Component	Standard implementation	Enhanced REP
Package	Intervention manual provided	Intervention manuals customized based on population needs (i.e., integrating trauma-informed approaches) and setting resources using input from the advisory board
Training	Standard training	Customized training based on input from package step above
Facilitation	As needed technical assistance with intervention delivery	**Facilitation components**: Review potential barriers and set goals based on barrier assessment; provide specific implementation guidance and facilitate information sharing and long-term plans for sustainability; meet regularly with providers; align intervention with organization, advocate for implementation to leadership

Previous research identifying key determinants of the MMH curriculum implementation established that teachers found the intervention was unable to consistently meet students’ needs (i.e., the context), in particular among students experiencing marginalization, trauma, and disadvantage, posed challenges to intervention acceptability, which, in turn, reduced fidelity [40]. While the teachers reported the curriculum is adaptable, they also reported that more intensive adaptations, including those designed to meet the needs of specific subgroups of students, were time and resource-intensive [41]. Teachers reported these adaptations were, however, critical and one teacher reported that “(the curriculum) turn(ed) students away, and it turned the group of students away who needed it most. The students who have substance abuse problems, have emotional/mental health problems [40].” Collectively, this research underscores the need for implementation strategies to facilitate the responsiveness of EBIs for those disproportionately at risk of marginalization, trauma, and ultimately substance misuse, abuse, and the development of substance use disorders [42, 43]. Deploying implementation strategies that incorporate systematic adaptations to improve intervention-context fit and needed training and support is a promising approach to enhancing intervention acceptability, and fidelity to ultimately achieving improved health outcomes. An important first step, however, is executing a pilot study designing and testing the feasibility of the implementation strategies; pilot studies of implementation strategies are underutilized and can provide vital information on designing and testing effective strategies through addressing potential design and methods uncertainties and assessing potential effects of the implementation strategy on implementation and student outcomes [44].

### Aims/objectives

The goal of the study is to design and test a multi-component implementation strategy (Enhanced REP) to enhance the effective delivery of a comprehensive health curriculum in community schools serving youth exposed to trauma and compare it to standard implementation.

#### Primary aims

The primary aim of this study will be to design and test Enhanced REP to deliver MMH. We will evaluate the feasibility, acceptability, and appropriateness of the implementation strategy, compared to standard REP (i.e., standard implementation) which will be the basis of a larger hybrid type 3 cluster-randomized trial.

The second primary aim is to conduct an economic evaluation to estimate implementation strategy costs and cost-effectiveness of Enhanced REP versus standard implementation to deliver MMH in preparation for a larger trial.

#### Secondary aims

The secondary aims are to evaluate the potential effectiveness of Enhanced REP versus standard implementation on youth outcomes including student drug use, drug use risk perceptions, quality-adjusted life years, and student-level fidelity (e.g., satisfaction, curriculum engagement). We will also assess fidelity to the MMH curriculum using a teacher dose delivered measure, consistent with previous MMH implementation research [27, 29, 45].

## Methods/design

### Sample and setting

This pilot study will include ten schools from two intermediate school districts (ISDs: provide general education and curriculum support to multiple school districts), or Regional Educational Service Agencies, in Michigan. In 2019, 20.5% of youth nationally and 22.0% in Michigan experienced 2 or more ACEs and the risk of exposure increased to 24.2% for youth who experienced economic hardship [46].

### Procedures

The implementation strategy will be deployed by the school health coordinators as they are key MMH implementation intermediaries. This is also consistent with current practices; school health coordinators work with schools across Michigan serving in 24 regional hubs to support health programming such as MMH [47]. The health coordinators maintain relationships with school districts and health teachers and provide support including training, technical assistance, and consultation for school health programs, practices, and policies [47]. Thus, by using existing infrastructure and capacity for deploying Enhanced REP to support teachers in delivering MMH with fidelity, we will enhance the likelihood of sustainment.

As a first step in designing Enhanced REP, we will convene an advisory board of stakeholders (e.g., school health coordinators, teachers, other school professionals, and admin). Given preliminary data findings and the rates of community-level trauma and drug use in the state, we will consult experts in trauma-informed approaches to support curriculum tailoring and advise on trauma-informed training options. We will work with the advisory board to identify key areas of the curriculum that can be tailored, that is, the form aspects of the intervention. We will distinguish these elements from the core elements needed to be retained to support MMH effectiveness. This process will be guided by systematic adaptation steps described by Escoffrey et al. [48] and the foundational theories (the health belief model and social cognitive theory) of the intervention, to ensure that we incorporate fidelity-consistent adaptations that will not compromise the curriculum effectiveness [23, 24, 49]. We will then integrate the proposed changes into the curriculum materials. This will include replacing out-of-date materials, incorporating trauma-informed mental and emotional health resources, making the format user-friendly and flexible for teachers, updating drug use information and resources, and adding new activities for students (e.g., online interactive activities). The advisory committee members will each receive $250 remuneration for participation in study activities.

The facilitation component will be based on the Quality Enhancement Research Initiative (QUERI) implementation facilitation strategy and iPARIHS framework [50, 51]. Facilitation promotes provider (i.e., teacher) capacity and self-efficacy in addressing barriers to MMH implementation [31]. The health coordinators will receive specialized training in facilitation based on the QUERI training program and a school-based trial deploying facilitation [31], adapted to Tier 1 prevention. Health coordinators will support teachers in implementing the tailored curriculum by engaging in the facilitation activities (see Table 2 for sample facilitation schedule and activities).

**Table 2:** Facilitation component of Enhanced REP, schedule of activities (adapted from Kilbourne et. al. [31])

Week(s)	Activity	Description
Week 1	Initiation and Benchmarking	The health coordinator will review the semester, provide an overview of the facilitation process, discuss implementation goals, and fit between goals and the teacher/classroom. He/she will identify potential barriers and facilitators and set broad goals for implementation.
Weeks 2–9	Mentoring	The health coordinator and teachers will have weekly phone or video meetings to develop rapport and guide teachers to address barriers to MMH implementation.
Weeks 3–10	Leveraging	The health coordinator will contact to school administration, identify school priorities per administrator input, and align the health curriculum with other school initiatives and priorities.
Ongoing	Marketing and sustainment	The health coordinator will work with administration, health teachers, and other school personnel as appropriate to develop a plan for ongoing implementation support and health curriculum alignment with other school initiatives.

The training component will include asynchronous and synchronous modules focused on trauma-informed approaches with teachers as the intended audience. Before conducting training, we will assess teachers’ exposure to and awareness of trauma-informed practices to tailor the training to meet their needs. Sample modules for the trauma-informed training component of the implementation strategy bundle are included in Table 3.

**Table 3:** Trauma-informed training component of Enhanced REP

Module	Type	Description	Methods
Module 1	Asynchronous	Foundational information about trauma-sensitive classrooms and trauma-informed approaches in education Largely based on existing evidence-based materials [50–53] Specific to trauma-informed adaptations made to the MMH curriculum and the rationale behind them	• Readings • Videos
Module 2	Synchronous	School professional resilience, secondary trauma, and retraumatization Opportunities to engage in active learning and practice applying concepts	• Applying concepts • Health coordinator input and support • Peer Q&A

Taking into consideration school closures and inconsistent education delivery in the 2020-2021 school year, we delayed the beginning of the study to September of 2021. The primary reason for this was to ensure conditions among intervention and control groups were as similar as possible and to decrease the likelihood that outcomes would be confounded by the learning modality of a school such as virtual compared to in-person learning. During this time, we planned to conduct user-testing of the tailored curriculum component of Enhanced REP. Two teachers who did not qualify to participate in the trial portion of the study tested the digital content and provided feedback. Following this user testing, the study team will meet with teachers and health coordinators to discuss feedback and identify areas for refinement prior to pilot study initiation.

**Fig. 2:**
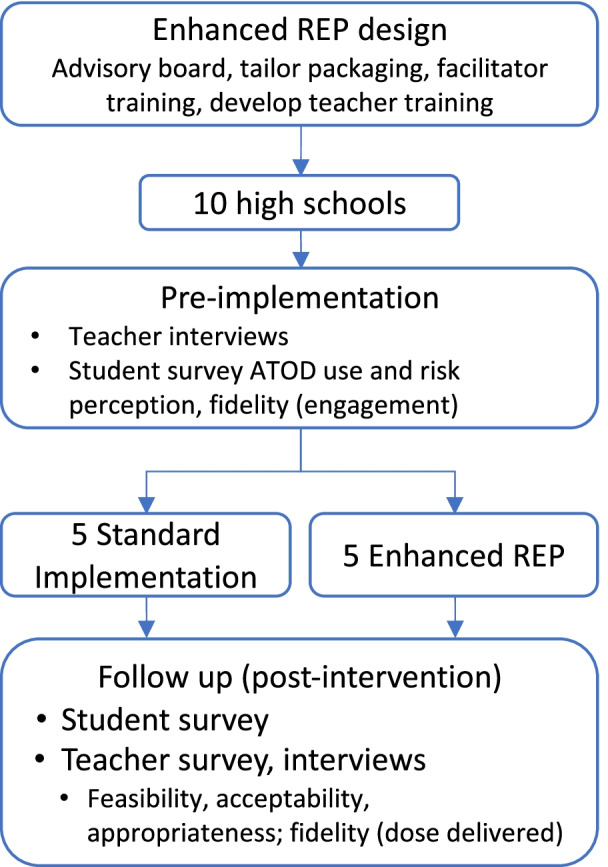
Group randomized controlled trial pilot study design. ATOD: alcohol, tobacco and other drugs

In cooperation with the school health coordinators, we will identify and recruit 10 high schools currently using MMH that fail to meet state standards for implementation (implementing less than 80% of the curriculum) and/or facing one or more barriers to MMH implementation, for participation. We will focus on schools where at least 20% of students are eligible for free and reduced lunch, as socioeconomic disadvantage is an additional risk factor for ACEs. We will match participating schools a priori on key characteristics such as school size and percent of students eligible for free/reduced lunch to ensure balance across study conditions [54]. We will randomize schools to either standard implementation (akin to standard REP) or Enhanced REP (see Table 1, Fig. 2). For the standard implementation condition, teachers will receive the MMH curriculum manual, standard training, and as-needed technical assistance, provided to them by the health coordinators. The Enhanced REP condition will receive an MMH curriculum manual tailored to incorporate trauma-informed approaches, tailored trauma-informed training, and implementation facilitation.

The health coordinators and study team will meet with teachers and administrators at participating schools to share study information. We will discuss specific procedures around data collection. Teachers will provide informed consent prior to the start of the study. We will conduct semi-structured interviews with teachers pre-implementation to assess readiness and post-implementation to assess the feasibility, acceptability, and appropriateness of standard implementation and Enhanced REP. Teachers will also complete a post-implementation survey to evaluate these constructs quantitatively. Students will be eligible for student-level surveys. Students will be recruited in conjunction with school district partners. The study team will communicate with teachers and administration, either in-person or using video conferencing and via email, to share study information.

The student survey questions will be similar to other school-based surveys (i.e., Mi-PHY: Michigan Profile for Healthy Youth), and we will follow a similar procedure for survey administration. The teachers will send a letter to parents to provide information and an opportunity to opt-out prior to the start of the study. Following parental letter and participant assent, students will complete a self-administered questionnaire through a secure, online server. As MMH is integrated as part of the school curriculum, students will complete the initial survey during their health class before the MMH delivery and at the end of the term. Students who do not assent will receive an alternate activity. We expect 300 students to participate, based on an 80% response rate across the schools, similar to other youth studies [55]. Each school will receive $500 to support their participation in the study and teachers will receive up to $300 per academic term as remuneration for study activities.

### Primary aim 1

Compare deploying Enhanced REP with standard implementation to deliver the MMH and evaluate the feasibility, acceptability, and appropriateness of the implementation strategy among teachers.

### Measures

#### Feasibility, acceptability, and appropriateness

**Fig. 3:**
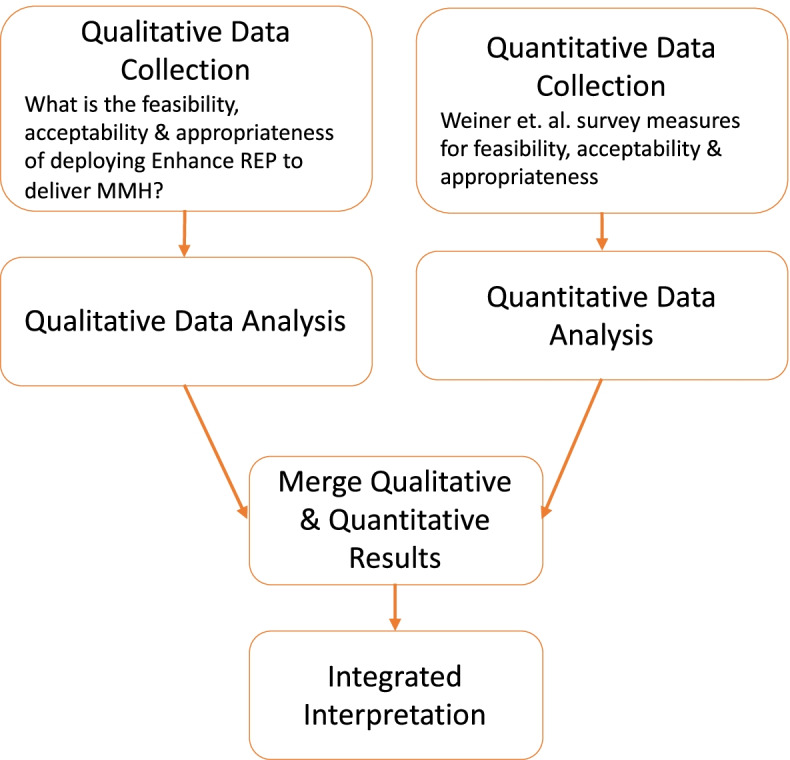
Aim 1 convergent mixed methods design (adapted from Creswell & Plano-Clark [58])

To evaluate comprehensively feasibility, acceptability, and appropriateness we will adopt a convergent mixed methods design (see Fig. 3). The purpose of a convergent design is to obtain “different but complementary data on the same topic [56]. We will use Weiner et al.’s [57] measures to assess acceptability, appropriateness, and feasibility. Each construct has four items (e.g., REP is appealing, REP seems suitable), using a 5-point Likert scale: 1—strongly disagree to 5—strongly agree. The interview guide will focus on eliciting feedback on specific Enhanced REP components (manual, training, and facilitation) and existing challenges with curriculum implementation.

#### Data analytic approach

#### Qualitative data

We will use an inductive/deductive thematic analytical approach outlined by Hsieh and Shannon for interview transcripts [59]. First, each member of the study team will review the transcript material to develop a broad understanding of the content [60]. Second, the empirical material contained in the interviews will be independently coded by project team members to condense the data into analyzable units. Segments of text ranging from a phrase to several paragraphs will be assigned codes based on a priori or emergent themes (also known as open coding; [61]). Codes will also be assigned to describe connections within and between categories and subcategories (axial coding; [61]). Third, the text will be independently coded by at least two study team members. Disagreements will be resolved through consensus and the team will develop a final codebook. Using this codebook, two study team members will separately review transcripts to determine the level of agreement in the codes applied [62]. Fourth, based on these codes, we will use qualitative software to generate a series of categories connecting text segments grouped into separate “nodes.” We will use these nodes to examine the association between different a priori and emergent categories. Fifth, the different categories will be further condensed into broad themes.

#### Quantitative data

We will evaluate appropriateness, acceptability, and feasibility using descriptive analyses from teacher surveys. The analyses will focus on descriptive statistics for the quantitative data from providers, including means, standard deviations, and proportions as appropriate.

#### Data integration

Results from each data set will be examined side-by-side to explore convergence (i.e., comparing analysis conclusions) to investigate if qualitative and quantitative results concur and complementarity (i.e., one set of findings elaborates on another). We will also investigate how interview results elaborate on quantitative results (expansion) to deepen our understanding of why and how Enhanced REP may or may not be acceptable, feasible, and appropriate and how this may influence fidelity [58]. Should discordant findings arise, we will use a method of managing such findings as described by Creswell and Plano Clark, such as collecting additional data, re-analyze existing datasets, and identifying potential sources of bias [58].

### Primary aim 2

Conduct an economic evaluation to estimate the costs and cost-effectiveness of deploying Enhanced REP versus standard implementation to deliver MMH.

#### Procedures

We will monitor the activities listed in Table 4 to estimate implementation strategy costs. The study team will track time for labor costs, which will constitute most of the resources/costs. This will include time logs for health coordinators to track Enhanced REP Facilitation activities, costs for training, and indirect costs associated with teacher time to attend tailored training and participate in implementation support. The study team will also track and compile all non-labor costs (e.g., adapted curriculum website updates and maintenance).

**Table 4:** Cost inputs for the economic evaluation of the Enhanced REP implementation strategy

Phase	Cost type	Description	Activities
Pre-Implementation	Intervention	Ongoing intervention costs	• Subscription to website to access health curriculum online • In-class materials: printing handouts, student assessments • Basic teacher training • Hard-copy of the curriculum (as indicated)
	Implementation	Prepare schools for implementation strategy deployment	• Time updating curriculum content (MDHHS^a^, health coordinator activity, community and/or academic partners) • Meet with school leadership, teachers, intermediate school districts (health coordinator activity) • Review logistics of Enhanced REP deployment (health coordinator/ISD^b^ activity) • Make needed modifications to website/material formats to fit with local school district (e.g., for Google Classroom; health coordinator activity) • Review Enhanced REP components (i.e., tailored curriculum) with participating schools and other stakeholders as indicated (health coordinator activity) • Review Enhanced REP components with appropriate experts in adaption are (e.g., trauma-informed approaches; health coordinator and community and/or academic partners)
Implementation phase	Intervention	Ongoing costs for delivering the MMH	• Intervention costs (subscription to regular online MMH curriculum) • Materials related to the curriculum (posters, etc.) • Printed materials for students (handouts, etc. as applicable)
	Implementation	Implementation strategy deployment	• Facilitation training for health coordinators (ISD activity) • Teacher training component of the Enhanced REP implementation strategy (health coordinator/ISD activity) • Meetings with teachers to set goals, expectations, engage in problem-solving to mitigate implementation barriers (i.e., facilitation; health coordinator activity) • Fidelity monitoring completed by teachers (teacher activity) • Travel-health coordinators visiting participating schools/classrooms (health coordinator activity)
Sustainment phase	Intervention		• Recurring costs for intervention subscription, updated materials, preparation time related to curriculum updates
	Implementation	Post-program evaluation	• Data analysis (school/district/ISD activity) • Implementation review (teacher/school activity/health coordinator) • Implementation strategy assessment (all levels) • Implementation strategy refinement (district/ISD/health coordinator activity) • Ongoing costs for implementation strategy deployment (e.g., time engaging in facilitation, teacher training) (school/district/ISD activity)

^a ^*MDHHS* Michigan Department of Health and Human Services, ^b ^*ISD* intermediate school district/regional school service agency

### Measures

#### Costs

Researchers have used micro-costing approaches frequently in implementation science [37]. One approach is a modified cost calculator approach that has been applied in the Costs of Implementing New Strategies (COINS); this approach identifies a range of costs across phases of implementation (e.g., pre-implementation, implementation, and sustainability) tailored to the strategies utilized for a specific implementation effort and focused on the perspective of the organization/provider deciding to adopt the EBI [63–66]. This is useful in identifying costs related to implementation for several reasons: (1) it aids in determining direct costs of implementation through tallying time spent on activities in each phase of implementation strategy deployment (often the bulk of implementation strategy costs), (2) this practical approach can provide needed guidance and scaffolding for stakeholders and decision-makers to determine implementation costs so organizations could accurately estimate the necessary resources for implementation success, and (3) this approach has been used previously with Enhanced REP in estimating costs as the first step in cost-effectiveness analysis for a community-based clinical trial [67]. Table 4 provides a list of anticipated activities whose costs will be estimated prospectively as part of the pilot trial. We will also assess the costs of the REP condition, or standard implementation of MMH, for the intervention materials and training using available data from MDHHS and the Michigan School Health Coordinators Association (MiSHCA). For the standard implementation condition, we will ask coordinators to track time spent on as-needed technical assistance.

#### Health outcomes

We will use the EQ-5D to assess Quality Adjusted Life Years (QALYs). The EQ-5D is a multi-attribute utility instrument that yields interval-level scores ranging from 0 (dead) to 1 (perfect health) [68]. This mapping provides a health utility measure for each health state experienced by patients in the study and can be used to calculate quality-adjusted life years, the preferred measure for health benefits used in cost-effectiveness analysis [52].

#### Data analytic approach

All costs will be adjusted to the current year's US dollars. Costs will include implementation strategy costs (i.e., inputs) listed in Table 4, such as labor costs for meetings, costs associated with training, and labor costs associated with the provision of facilitation. We will estimate the costs of Enhanced REP and standard implementation using the cost data, with the comparator strategy being standard implementation. Our primary utility will be a change in reported quality-adjusted life years (QALYs). We will also assess changes in student drug use during the intervention period. We will use net costs (net increase in costs from Enhanced REP compared to standard implementation) and net effectiveness (net change in drug use for Enhanced REP versus standard implementation) to estimate the incremental cost-effectiveness ratio for student outcomes. We will conduct a one-way sensitivity analysis on all cost input parameters listed in Table 4 as well as health utilities to provide estimates of the costs and incremental cost-effectiveness to decision-makers. The analysis will also include multi-way sensitivity analyses on the parameters whose results are most sensitive in influencing the cost-effectiveness ratio [52]. This will inform the feasibility of our costing approach, provide preliminary information on cost input distributions and which inputs may be especially influential on cost-effectiveness ratios to inform the detail of future data collection efforts; this pilot study will also provide preliminary information on the cost-effectiveness of Enhanced REP to inform the utility of undertaking a CEA in a larger trial [53].

### Secondary aims

Our secondary aim is to evaluate the potential effectiveness of Enhanced REP versus standard implementation on student outcomes. Student outcomes include drug use, drug use risk perceptions, and student-level fidelity (e.g., satisfaction, curriculum engagement) as described by Barrera et al. [69]. We will also assess MMH fidelity using the teacher dose delivered.

### Measures

Secondary aim study measures are summarized in Table 5.

**Table 5:** Secondary outcome measures

Measures	***# of items***	***Scaling***	***Timing***	***Source***
**Behavioral outcomes**				
Marijuana, cigarette, e-cigarette, alcohol, binge drinking	10	1 = none; 7 = 40 or more times	Pre- and post-implementation	MTF [70] adapted
Prescription drug misuse (incl. frequency, motivation, diversion)	12	1 = none;7 = 40 or more times, 18 options; choose all that apply	Pre- and post-implementation	MTF [70] adapted
Other substance use (poly-drug)	2	1 = none; 7 = 40 or more times	Pre- and post-implementation	MTF [70], NSDUH [71] adapted
Drug use risk perceptions	10	1 = no risk; 5 = great risk	Pre- and post-implementation	MTF [70]
**Implementation outcomes**				
Fidelity engagement				
Satisfaction	4	1 = strongly disagree; 5 = strongly agree	Post-implementation	Giles et al. [72] adapted
Key skills (assertive communication, refusal skills, decision making)	9	1 = strongly disagree; 5 = strongly agree	Post-implementation	National Health Education Standards [26], MMH [73]

#### Behavioral outcomes

We will assess past 3- and 12-month substance use using items from Monitoring the Future (MTF; [70]) with adapted response options and timeframe.

#### Implementation outcomes, student-level

##### Fidelity: engagement

Engagement has been identified as a fidelity dimension that provides information on participant responsiveness to the intervention and is key to intervention success [69]. As described in Barrera et al. [69], we will assess engagement using student satisfaction and key intervention skills. The satisfaction measure will be adapted based on a scale developed by Giles et al. for another drug prevention intervention with good psychometric properties that will include four items [72]. We will evaluate key intervention skills: assertive communication, refusal skills, and decision-making. These dimensions are identified in the MMH curriculum summative evaluation materials, assessed in previous MMH studies, and based on National Health Education Standards [21, 26]. Students will rate their level of agreement using a 5-point Likert scale on their proficiency with specific elements related to each skill.

#### Fidelity, teacher-level: MMH dose delivered

We will assess the dose or amount of the intervention delivered using a curriculum fidelity tracking form. Teachers will complete a brief form following each lesson and unit included in the study. Lessons are grouped into multiple units, including the alcohol, tobacco, and other drug prevention unit, the skills unit, and the social and emotional learning unit. We will assess the dose delivered by calculating the total number of lessons completed within each unit (10 lessons/unit). We will also ask teachers to report any adaptations or modifications, guided by the framework proposed by Wiltsey-Stirman et al. [74] on the tracking form. Modifications include adding, removing, and changing content, substituting activities, and changing activity formats.

### Data analysis

#### Secondary outcomes

The focus of this pilot trial will be to generate information to inform our approach to collecting student-level data and estimate the treatment effects variance estimates for a larger study [75]. We will calculate and examine treatment effects using linear mixed-effects models (LMM), with an understanding of potential challenges with estimating treatment effects for pilot studies [76]. LMM are appropriate models for analyzing clustered data and may involve fixed and random effects [77]. We will control for key demographic variables in all analyses, including race/ethnicity gender, and socioeconomic status.

We will assess MMH dose delivered using the sum score across all units as well as unit-specific dose delivered calculations. Consistent with previous research, we will also calculate the proportion of the units delivered, individually and combined, with a focus on descriptive statistics (means, standard deviations).

## Discussion

This research has the potential to support the design and deployment of effective and sustainable strategies for implementing drug use prevention interventions in schools. Preventing or tempering the onset and escalation of drug use can reduce the burden of social, emotional, and economic costs placed on youth and their families, communities, and society [3]. The proposed research can contribute to improving public health and reducing health disparities related to the disparate consequences of drug use and addiction among youth exposed to trauma. Systematically designing and testing implementation strategies to address key determinants implementation, including poor fit between the intervention and context/population needs, will support refinement and ultimately, leadership engagement in ongoing implementation support once the research study ends. Economic evaluation can provide vital information for stakeholders and decision-makers regarding implementation strategies to support and sustain quality EBI delivery*.*

This project has several strengths. First, we will design and systematically deploy a theoretically grounded bundle of implementation strategies, Enhanced REP, to address key determinants. Second, researchers have noted that many full implementation trials have progressed without initial pilot trials [44]; thus, this research will support the quality and rigor of full implementation trials by providing an opportunity to refine the implementation strategy and address issues around the feasibility of research methods to enhance the contributions to the field [44]. This research has the potential to support the advancement of research-to-practice translation of substance use prevention programs through designing implementation strategies that are effective, sustainable, and that improve quality program delivery. Third, we will use a community-engaged approach to designing the implementation strategy by incorporating an advisory board composed of multiple stakeholders. Fourth, we will estimate the costs and cost-effectiveness of the implementation strategies versus standard implementation to help address a central challenge to the effective implementation and sustainment of EBIs. This has the potential to provide useful, accessible information for communities to make well-informed decisions about resource allocation and implementation. Fourth, this project has the potential to advance implementation strategies for universal substance use prevention interventions. Researchers have estimated that effectively implementing school-based prevention would save an estimated lifetime monetary cost of $33.5 billion and a total cost of $98.6 billion to society for early adolescents using costs estimated in 2002 [78]. Finally, this research will support using implementation science to meet the needs of underserved families and communities who are at increased risk of trauma exposure, drug use, and its consequences.

We also note several potential study limitations. While the study will work with schools serving under-resourced families across two counties in Michigan, schools are heterogeneous settings with different organizational structures. Thus, different schools may experience other relevant barriers at various levels not addressed in the Enhanced REP strategy bundle. Alternative strategies may be needed to support successful implementation, including strategies in the outer setting, and may be an important focus of future research. We did not include students explicitly in the implementation strategy design process. Although implementation strategies often focus on the intervention deliverer and relevant contextual considerations, incorporating the input of the intervention recipients in addition to assessing their responsiveness (e.g., engagement) is an important future direction and potential focus of a larger trial.

The next step in this program of research would be to test the implementation strategy on a larger scale over a longer duration, including a large-scale cluster-randomized trial Type 3 hybrid trial. This would be conducted in schools serving youth experiencing ACEs and other forms of adversity across multiple states adopting the MMH curriculum. This research will also support scaling up these strategies to improve health curriculum delivery and advance substance use prevention.

## Data Availability

Electronic copies of publications will be made accessible to a journal in PubMed Central. This article is licensed under the Creative Commons Attribution 4.0 Generic License (CC BY 4.0), which permits use, sharing, adaptation, distribution, and reproduction if appropriate credit to the original author(s) is provided along with a link to the Creative Commons license and an indication of changes. De-identified primary participant (i.e., student)-level data will be available through an appropriate data repository, such as the NIH HEAL (National Institutes of Health: Helping to End Addiction Long-term^SM^) Initiative central data repository. This data will be available upon acceptance for publication of the main findings from the final student-level dataset. Data will be available in the NIH HEAL repository per HEAL guidelines. Access to individual-level data will require entering into a data-sharing agreement that includes requirements to protect participants’ privacy and data confidentiality.

## Abbreviations

ACEs: Adverse childhood experiences
CASEL: Collaborative for Academic, Social, and Emotional Learning
CDC: Centers for Disease Control and Prevention
COINS: Costs of Implementing New Strategies
DSMB: Data Safety and Monitoring Board
EBI: Evidence-based intervention
Enhanced REP: Enhanced Replicating Effective Programs
HIPAA: Health Insurance Portability and Accountability Act
iPARIHS: Integrated Promoting Action on Research Implementation in Health Services
IRB: Institutional Review Board
ISDs: Intermediate School Districts
LMM: Linear mixed-effects models
Mi-LEAP: Michigan Model for Health^TM^: Learning to Enhance and Adapt for Prevention
MMH: Michigan Model of Health^TM^
MTF: Monitoring the Future
NIDA: National Institute on Drug Abuse
NIH HEAL: National Institutes of Health Helping to End Addiction Long-term^SM^
NSDUH: National Survey on Drug Use and Health
QALYs: Quality-adjusted life years
QUERI: Quality Enhancement Research Initiative
SUD’s: Substance use disorders
